# Myrf guides target gene selection of transcription factor Sox10 during oligodendroglial development

**DOI:** 10.1093/nar/gkz1158

**Published:** 2019-12-12

**Authors:** Jessica Aprato, Elisabeth Sock, Matthias Weider, Olga Elsesser, Franziska Fröb, Michael Wegner

**Affiliations:** Institut für Biochemie, Emil-Fischer-Zentrum, Friedrich-Alexander-Universität Erlangen-Nürnberg, Erlangen, Germany

## Abstract

Oligodendrocytes generate myelin in the vertebrate central nervous system and thus ensure rapid propagation of neuronal activity. Their development is controlled by a network of transcription factors that function as determinants of cell identity or as temporally restricted stage-specific regulators. The continuously expressed Sox10 and Myrf, a factor induced during late development, are particularly important for terminal differentiation. How these factors function together mechanistically and influence each other, is not well understood. Here we show that Myrf not only cooperates with Sox10 during the induction of genes required for differentiation and myelin formation. Myrf also inhibits the activity of Sox10 on genes that are essential during earlier phases of oligodendroglial development. By characterization of the exact DNA-binding requirements of Myrf, we furthermore show that cooperative activation is a consequence of joint binding of Sox10 and Myrf to the same regulatory regions. In contrast, inhibition of Sox10-dependent gene activation occurs on genes that lack Myrf binding sites and likely involves physical interaction between Myrf and Sox10 followed by sequestration. These two opposite activities allow Myrf to redirect Sox10 from genes that it activates in oligodendrocyte precursor cells to genes that need to be induced during terminal differentiation.

## INTRODUCTION

In the vertebrate central nervous system (CNS), oligodendrocyte-dependent myelination ensures rapid saltatory conduction along axons. If myelin is defective or damaged, severe cognitive and motor disabilities result. Oligodendrocytes acquire their ability to form myelin sheaths around axonal segments during terminal differentiation from committed oligodendrocyte precursor cells (OPCs).

This process is regulated by a complex regulatory network that is constructed around central transcriptional regulators and additionally includes chromatin modifying proteins and regulatory RNAs (1–4). Among transcriptional regulators, the bHLH domain containing Olig2 and the HMG domain containing Sox10 are particularly important as they are present at all times of oligodendroglial development. They simultaneously determine oligodendroglial identity and stage-specific expression patterns. As a consequence, expression of some target genes will be regulated by these factors at all times, whereas others will be under their control only during specific developmental phases (5).

Many genes that are activated during terminal differentiation in oligodendrocytes and required for myelination have been identified as direct target genes of Sox10 (6). It has furthermore been shown that Sox10 is helped in its function by Myelin Gene Regulatory Factor (Myrf), a transcription factor that becomes expressed shortly before terminal differentiation and is itself a Sox10 target gene in these cells (7,8). Myrf is a large protein with an immunoglobulin-type Ndt80 domain for DNA-binding in its aminoterminal region, an intramolecular chaperone domain (ICD) for trimerization and autoproteolysis in the central portion and a transmembrane-domain in its carboxyterminal part that anchors the protein in the membrane of the endoplasmic reticulum (ER) (9,10). Upon homotrimerization and autoproteolysis, the trimerized aminoterminal half is released, enters the nucleus and supports Sox10 in the induction of the myelination program.

In contrast to the large number of validated target genes of Sox10 in differentiating oligodendrocytes, there are only few known Sox10 target genes in OPCs. Some evidence has been recently obtained for *Pdgfra* and *NG2/Cspg4* as potential targets (11,12). Pdgfra, in particular, is highly relevant as it determines proliferation, survival and migration of OPCs downstream of platelet derived growth factor (Pdgf).

Considering the existence of stage-specific target genes for Sox10 during oligodendroglial development, mechanisms must be in place that temporally restrict Sox10 activity on the corresponding regulatory regions and direct it from one set of target genes to another. The selective occurrence of cooperating factors such as Myrf in differentiating oligodendrocytes represents one important mechanism (8). Additionally, there is evidence that proteins in OPCs such as Hes5 and SoxD factors prevent Sox10 from activating genes that it targets later during terminal differentiation (13,14). However, no mechanism has yet been described that explains the selective downregulation of those Sox10 target genes that are expressed in OPCs and whose expression needs to be extinguished in differentiating oligodendrocytes.

To increase knowledge of Sox10 target genes in OPCs and the mechanisms by which their expression is temporally restricted, we combined results from RNA-Seq and ChIP-Seq studies to define a large number of OPC-specific Sox10 target genes and then analyzed several of these target genes to understand on a mechanistic level how their expression is turned off in differentiating oligodendrocytes despite the continued presence of Sox10. Our study identified Myrf as a decisive factor that helps Sox10 to switch between its target genes. Our analyses also revealed hitherto unknown and unexpected features of Myrf function.

## MATERIALS AND METHODS

### Cell culture

Primary oligodendroglial cells were obtained from newborn Wistar rats of both sexes after growth in mixed glial cultures by shake-off (15). Oligodendroglial cells were grown on poly-ornithine substrate under proliferative conditions in serum-free SATO medium containing N2 supplement, 10 ng/ml Pdgf-AA and 10 ng/ml Fgf2. Differentiation was induced by replacing the mitogens by 1% FCS (16). In some experiments, oligodendroglial cells were retrovirally transduced at a moiety of infection of 0.2 under proliferative conditions.

Spontaneously immortalized rat OLN93 oligodendroglial cells (gift of C. Richter-Landsberg) (17), mouse N2a neuroblastoma cells (obtained from ATCC, #CCL-131) and human embryonic kidney 293 (HEK293) cells (obtained from ATCC, #CRL-1573) were grown in DMEM supplemented with 10% fetal calf serum (FCS). Of the cell lines, only OLN93 cells were authenticated by PCR. None was checked for mycoplasma contamination. N2a cells were used for luciferase reporter assays (8), HEK293 cells for preparation of protein extracts (18). Genome-editing of OLN93 cells and characterization of resulting cell clones has been described elsewhere in detail (19).

### RNA-Seq and bioinformatic analysis

Total RNA was prepared from independent OLN93 cell clones A and B that had undergone CRISPR/Cas9-dependent genome editing to inactivate Sox10 with different guide RNAs and two control clones using the RNeasy Micro Kit (Qiagen). RNA samples were treated with DNase I to remove contaminating DNA. Quality and purity of samples were evaluated using an Agilent 2100 Bioanalyzer (Agilent Technologies Germany). 100 ng total RNA were used for library preparation (Ilumina Stranded mRNA Kit). Approximately 52 million reads were generated per library using an Illumina Hiseq 2500 platform sequencer (Next Generation Sequencing Core Facility, FAU Erlangen-Nürnberg) and mapped onto rat genome rn5 using STAR (version 2.5.1.b). Unique mappings were detected using HTSeq count based on ENSEMBL Gene identifier Version 75. Statistical analysis was carried out using DESeq2 R Version 1.8.1. Gene expression values are deposited in GEO under accession number GSE136659.

To identify direct Sox10 target genes in oligodendroglial cells, genes were summarized in a list that were downregulated in Sox10-negative OLN93 cells (≤–2-fold, *P* ≤ 0.05). A second list was generated that contained all genes associated with CNS-specific Sox10 ChIP-Seq peaks (GSE64703) (20) as determined by intersect (version 1.0.0). The two gene lists were then compared using the Venn webtool on the BEG homepage (http://bioinformatics.psb.ugent.be/webtools/Venn/). The resulting 203 genes with Sox10-dependent expression and nearby genomic Sox10 binding site were defined as potential Sox10 target genes. Using lists of genes with preferential expression in OPCs or differentiating oligodendrocytes (21) and comparing them to the 203 genes using the Venn webtool, potential Sox10 target genes were classified as enriched in OPCs or in differentiating oligodendrocytes. Gene ontology (GO) analysis of OPC-enriched direct Sox10 target genes was performed using the Gene Ontology enrichment, analysis and visualization tool (http://cbl-gorilla.cs.technion.ac.il/) in combination with semantic clustering by REViGO (http://revigo.irb.hr/) (22). The Integrative Genomics Viewer (IGV) was used to visualize select ChIP-Seq peaks.

### Plasmids and viral constructs

**Figure 1.**
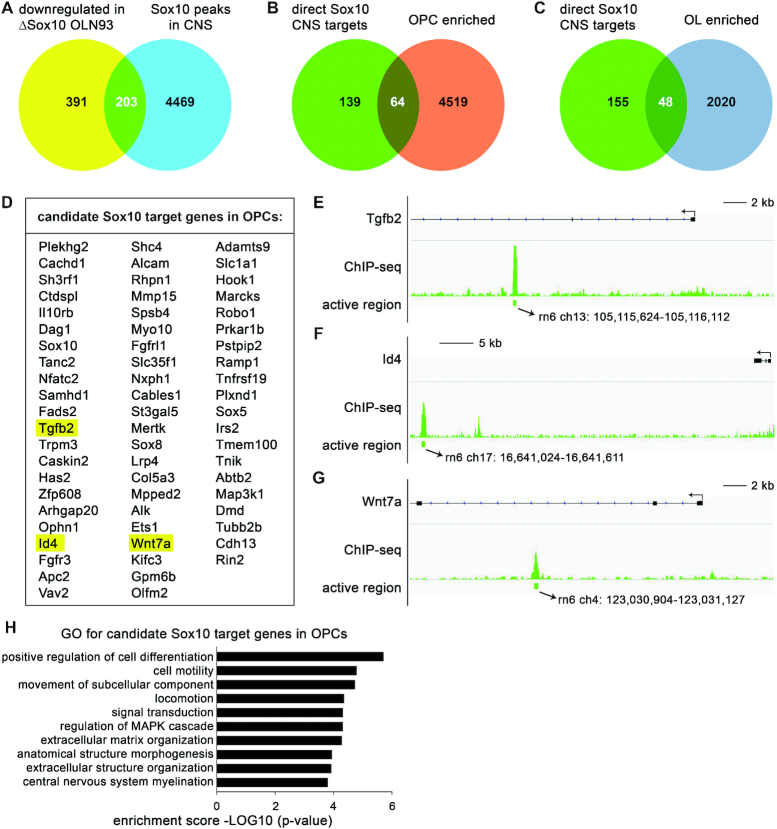
Identification of OPC-specific target genes of Sox10. (**A**) Venn diagram identifying 203 candidate oligodendroglial target genes of Sox10 as the overlap between genes differentially downregulated (≤–2-fold; *P* ≤ 0.05 according to GSE136659) in OLN93 cells following Sox10 inactivation (ΔSox10 OLN93) and genes exhibiting Sox10-binding in their vicinity in the CNS (20). (**B, C**) Venn diagram depicting the fraction of oligodendroglial target genes of Sox10 with preferential expression in OPCs (B) or differentiating oligoendrocytes (C) as determined by analysing the stage-specific oligodendroglial expression pattern (21) for the potential target genes. (**D**) List of 64 candidates for Sox10 target genes in OPCs. Genes with strongest downregulation in Sox10-deficient OLN93 cells are at the top of the list. (**E–G**) IGV tracks showing Sox10 binding sites in the vicinity of the *Tgfb2, Id4* and *Wnt7a* genes according to ChIP-Seq studies (20). Exact chromosomal locations in the rat genome (rn6) are given below the tracks. Size bars correspond to 2 or 5 kb as indicated. (**H**) Biological terms associated with OPC-specific Sox10 target genes according to gene ontology (GO) studies. Ranking was by enrichment score. Genes listed under the terms were: Fgfr3, Dmd, Sox8, Tnik, Plxnd1, Olfm2, Adamts9, Ets1, Tgfb2, Marcks, Id4, Sox10, Nfatc2, Robo1, Has2, Tmem100, Sox5, Tubb2b, Dag1 for positive regulation of cell differentiation; Sox8, Plxnd1, Ets1, Tgfb2, Wnt7a, Cdh13, Sox10, Apc2, Nfatc2, Robo1, Mertk, Vav2, Sh3rf1, Tubb2b, Pstpip2 for cell motility and for locomotion; Sox8, Plxnd1, Ets1, Tgfb2, Wnt7a, Cdh13, Sox10, Apc2, Nfatc2, Robo1, Kifc3, Mertk, Vav2, Sh3rf1, Tubb2b, Alcam, Dag1, Pstpip2 for movement of subcellular component; Ramp1, Fgfr3, Alk, Dmd, Plxnd1, Myo10, Prkar1b, Adamts9, Tgfb2, Cdh13, Wnt7a, Ophn1, Apc2, Robo1, Il10rb, Vav2, Fgfrl1, Tmem100, Map3k1, Arhgap20, Irs2, Tnik, Sox8, Rin2, Spsb4, Lrp4, Shc4, Nfatc2, Mertk, Rhpn1 for signal transduction; Tnik, Tnfrsf19, Tgfb2, Wnt7a, Sh3rf1, Map3k1 for regulation of MAPK cascade; Has2, Mmp15, Adamts9, Tgfb2, Gpm6b, Dag1, Col5a3 for extracellular matrix organization and for extracellular structure organization; Fgfr3, Dmd, Tnik, Sox8, Plxnd1, Lrp4, Adamts9, Tgfb2, Cdh13, Wnt7a, Id4, Sox10, Ophn1, Robo1, Has2, Mertk, Fgfrl1, Map3k1, Dag1 for anatomical structure morphogenesis; Fgfr3, Id4, Sox10, Dag1 for central nervous system myelination.

**Figure 2.**
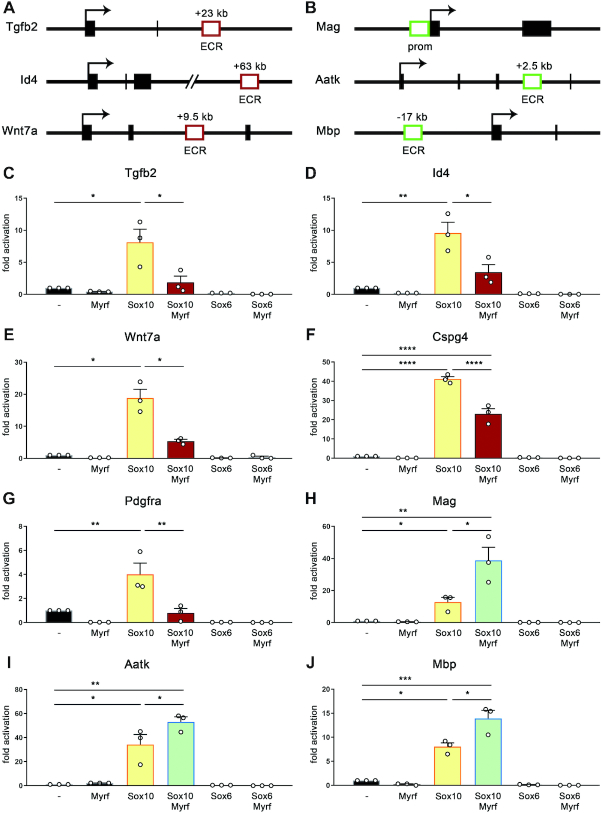
Effects of Myrf on the activity of regulatory regions of OPC-specific and oligodendrocyte-specific target genes of Sox10. (**A, B**) Localization and distance of ECRs (open boxes) relative to the TSS (arrow) of the mouse *Tgfb2, Id4*, *Wnt7a*, *Mag, Aatk* and *Mbp* genes. Exons are depicted as black boxes. In case of *Mag*, the ECR corresponds to the promoter (prom). (**C–J**) Luciferase assays in N2a cells transiently transfected with reporter genes under control of regulatory regions from the *Tgfb2* (C), *Id4* (D), *Wnt7a* (E), *Cspg4* (F), *Pdgfra* (G), *Mag* (H), *Aatk* (I) and *Mbp* (J) genes in the absence (–) or presence of Myrf, Sox10, Sox6 or a combination of Myrf and Sox protein. Reporter gene expression was determined in extracts 48 h after transfection and effector-dependent activation rates are presented as fold inductions ± SEM with transfections in the absence of effectors arbitrarily set to 1 for each reporter construct (*n* = 3–4). Differences were statistically significant as determined by one way Anova with Bonferroni correction (*P ≤ 0.05; **P ≤ 0.01; ***P ≤ 0.001).

Regulatory regions from the *Id4*, *Tgfb2*, *Wnt7a* genes (650–1000 bp, for localization, see Figure 1E–G and 2A) were obtained by PCR from mouse genomic DNA and inserted into the pTATAluc reporter plasmid (23). In case of the pTATAluc reporter containing the *Wnt7a* regulatory region, an additional version was generated with inserted synthetic Myrf binding site (c)3(c) (Figure 5A). The pTATAluc plasmid was also used to place the Sox10 binding site C/C’ (23) in front of the β-globin minimal promoter to generate the dimluc reporter construct. Into the dimluc reporter potential Myrf binding sites were additionally inserted (for sequences, see Figures 5A and 6A). Luciferase reporter plasmids with regulatory regions of the *Pdgfra*, *Cspg4*, *Mag, Aatk* and *Mbp* genes were as described before (8,12,19,24). Site-directed mutagenesis of Myrf binding sites (see Figure 7A) was performed with the Q5^®^ Site-Directed Mutagenesis Kit (New England Biolabs).

Myrf variants (see Figure 4A) were generated for this study by PCR from a full-length mouse Myrf cDNA or have already been described (25). They were inserted into pCMV5 expression vectors. For MyrfΔC, an additional version was generated with myc epitope at its aminoterminal end. pCMV5-based eukaryotic expression plasmids for full-length Myrf, Sox6 and Sox10 were as reported (8,14,18). Full-length Myrf and the MyrfΔC variant were also inserted behind the chicken β-actin (CAG) promoter into a retroviral CAG-IRES-GFP vector (26) to generate Myrf overexpressing retroviruses. Myrf expressing and control retroviruses co-expressed GFP for visualization of transduced cells.

### Luciferase assays, extract preparation, electrophoretic mobility shift assays and protein interaction studies

For luciferase assays, N2a cells were transfected with 0.5 μg of luciferase reporter and 0.5 μg of pCMV5-based expression plasmid per 3.5 cm plate using Superfect reagent (Qiagen). Overall amounts of plasmid in a particular experiment were kept constant by adding empty pCMV5 where necessary. Whole cell extracts were prepared 48 h after transfection by lysing cells in buffer containing 0.1% Triton X-100 and 2.5 mM ATP. Luciferase activities were determined after addition of luciferin substrate by chemiluminescence.

For preparation of protein extracts, HEK293 cells were transfected on 10 cm plates with 10 μg of pCMV5-based expression plasmids using polyethylenimine. 48 h after transfection, whole cell extracts were prepared by using 1% NP-40 to initiate cell lysis and 400 mM NaCl to extract nuclear proteins (27).

Whole cell extracts were used in electrophoretic mobility shift assays (EMSA) or protein interaction studies. EMSA were performed with extracts and ^32^P-labeled double-stranded oligonucleotides containing putative Myrf binding sites (for sequences, see Figures 5A and 6A, B). Oligonucleotides were 33–41 bp in length. PolydIdC was used as unspecific competitor at a final concentration of 0.1 μg/μl.

For co-immunoprecipitation experiments, HEK293 cell extracts were incubated with rabbit antibodies against Sox10 (home made) (28) or mouse monoclonals against the myc epitope (Cell Signaling Technology clone 9B11, 1:10000 dilution) and protein A sepharose beads (GE Healthcare). For GST-pulldown assays, whole cell extracts of transfected HEK293 cells were incubated with bacterially expressed and purified GST or GST-Sox10 fusion proteins bound to glutathione sepharose beads (27). After extensive washing, bead-bound material was eluted by boiling in 150 mM Tris–HCl, 6% SDS, 15% β-mercaptoethanol, 30% glycerine, 0.3% bromophenol blue and compared to the extracts used as input after size separation on 10% SDS-polyacrylamide gels by Western blotting. The following primary antibodies and detection reagents were used: rabbit anti-Sox10 antiserum (1:3000 dilution), mouse anti-myc monoclonal (clone 9B11, 1:10 000 dilution) and horseradish peroxidase coupled to protein A (Zymed, #10-1023, 1:3000 dilution). Detection was by chemiluminescence using ECL reagent. Images of Western blots have been cropped for presentation.

### Chromatin immunoprecipitation (ChIP)

Chromatin was prepared from rat oligodendroglial cells cultured three days under differentiating conditions. Chromatin was cross-linked with 1% paraformaldehyde and sheared to fragments of ∼200–400 bp in a Bioruptor (Diagenode) (27). After pre-clearing, chromatin was incubated with rabbit antiserum against Myrf (home made, raised against epitopes in the aminoterminal 386 amino acids) (8), rabbit antiserum against Sox10 (28), or preimmune sera before addition of protein A sepharose beads and precipitation. Crosslinks in precipitated chromatin were reversed and DNA was purified by proteinase K treatment, phenol/chloroform extraction and ethanol precipitation. Detection and quantification of the regulatory regions from the *Tgfb2, Id4*, *Wnt7a*, *Mag*, *Aatk* and *Mbp* genes in precipitated DNA was by qPCR on a Bio-Rad CFX96 thermocycler with each reaction performed in triplicates. The ΔΔCt method was used to calculate the percent recovery of a given DNA segment relative to the total input. After normalization, the enrichment of chromatin in the various precipitates over the input was determined. The sample with highest enrichment in a specific experiment was set to 1 and all other values were expressed relative to it.

The following primers were used for the detection of specific DNA regions: 5′-AAGTTCACATTCAGCGAAACG-3′ and 5′-GAGGACCCCTGAGTGAACAA- 3′ were used to amplify the *Tgfb2* regulatory region, 5′-GATAAAGGAAGGCTTTCAGCAA-3′ and 5′-TTGGATGGTGTGGTGCAG -3′ to amplify the *Id4* regulatory region, 5′-GAGCGAAAATCCAGGATGAA-3′ and 5′-GGATGGATGGGAAGTCCTTT-3′ to amplify the *Wnt7a* regulatory region, 5′- TTGGATGGTCTGGCTTCTG-3′and 5′-CCCATCTTCTCCAGGAAGG-3′ to amplify the *Mag* promoter region, 5′-AGCAAGAAGCTGGTGCTGAG-3′and 5′- GAGTGGGTGCAGTGAGTCCT-3′ to amplify the *Aatk* regulatory region, 5′-TTTGCTCACTCGAAGGGACT-3′and 5′-TTAGGTCCTTCTGGGGACAGT-3′ to amplify the *Mbp* regulatory region, and 5′-AGAGACTGGTTGCCAGGAAG-3′ and 5′-GGTGGAGACAGACTCGGAAC-3′ to amplify a negative control region (NCtrl).

### Immunocytochemistry

Primary oligodendroglial cells were cultivated for two days under proliferative conditions or for 6 days in differentiation medium on cover slips before fixation in 4% paraformaldehyde for 10 min. In some experiments, BrdU was added at a final concentration of 4 mM to proliferation medium 2 h before fixation. Cells on cover slips underwent immunocytochemistry using the following primary antibodies: rat anti-Mbp monoclonal (Bio-Rad, #MCA409S, 1:750 dilution), guinea pig anti-Sox10 antiserum (home made, 1:5000 dilution) (29), rabbit anti-Id4 (Novus Biologicals NBP2-56322, 1:50 dilution), mouse anti-Tgfb2 (Abcam ab36495, 1:50 dilution) and mouse anti-Wnt7a (Santa Cruz Biotechnology, E-9: sc-365665, 1:50 dilution). Secondary antibodies were coupled to Cy3 (Dianova, 1:400 dilution), Cy5 (Dianova, 1:400 dilution) or Alexa Fluor 488 (Molecular Probes, 1:0 000 dilution) fluorescent dyes. Incorporated BrdU was visualized using rat anti-BrdU (Abcam ab6326, 1:200 dilution). Stainings were documented with a Leica DMI6000 B inverted microscope (Leica) equipped with a DFC 360FX camera (Leica).

### Quantifications and statistical analysis

Results from independent experiments were treated as biological replicates. Sample size was *n* ≥ 3 for all molecular biology experiments and experiments using cell cultures as common for this kind of study. No data were excluded from the analysis. GraphPad Prism6 (GraphPad software, La Jolla, CA, USA) was used to determine whether differences in cell numbers, luciferase activities or immunoprecipitated DNA were statistically significant by one way Anova with Bonferroni correction or two-tailed Student's *t* tests (*P ≤ 0.05; **P ≤ 0.01, ***P ≤ 0.001). Variance between statistically compared groups was similar.

## RESULTS

### Sox10 activates a specific set of genes in OPCs

We have previously used CRISPR/Cas9-dependent genome editing to inactivate Sox10 in the immortalized rat oligodendroglial cell line OLN93 (19). Here we performed RNA-Seq on independent clones and compared the expression profile to OLN93 control clones. Among the 594 genes that were reproducibly downregulated ≥2-fold, 203 had previously been shown by ChIP-Seq to have closely associated genomic Sox10 binding sites in oligodendroglial cells of the CNS (20) (Figure 1A). By comparing the 203 genes with published lists of genes enriched in OPCs or differentiating oligodendrocytes from expression profiling data (21), 64 were identified as being preferentially expressed in OPCs relative to differentiating oligodendrocytes (Figure 1B), whereas 48 were found to exhibit higher expression in differentiating oligodendrocytes than in OPCs (Figure 1C). Expression of the remaining 91 genes did not differ substantially during oligodendrocyte differentiation. The 64 genes that have Sox10 binding regions near or within them, depend on Sox10 for their expression and occur preferentially in OPCs, are excellent candidates for OPC-specific Sox10 target genes (Figure 1D–G). Gene ontology studies indicated that the genes are associated with regulation of cell differentiation, cell motility and extracellular matrix/structure organization indicating that Sox10 may influence lineage progression, migration and production of secreted proteins in OPCs (Figure 1H).

### OPC-specific and oligodendrocyte-specific Sox10 target genes respond differentially to Myrf

Among the potential OPC-specific Sox10 target genes, *Tgfb2, Id4* and *Wnt7a* caught our attention (Figure 1D). Tgfb2 has been previously shown to determine the migratory properties of OPCs and their relationship with neurons (30). Id4 helps OPCs to maintain their progenitor state and interferes with oligodendrocyte differentiation (31–33), and Wnt7a regulates the crosstalk of OPCs with vasculature and neurons (34).

ChIP-Seq peaks for Sox10 in the vicinity of these genes (Figure 1E–G) (20) were found to localize in evolutionary conserved regions (ECRs) 23 kb downstream of the transcription start site (TSS) of the *Tgfb2* gene, 63 kb downstream of the TSS of the *Id4* gene, and 10 kb downstream of the TSS of the *Wnt7a* gene in the mouse genome (Figure 2A).

The three identified ECRs were inserted in luciferase plasmids to study their Sox10 responsiveness in reporter gene assays. For comparison, plasmids containing the *Pdgfra* promoter or an intronic enhancer of the *Cspg4* gene were also tested as these two regions represent the only other regulatory regions known to be under control of Sox10 in OPCs (12,24). Additionally, we included luciferase plasmids containing the promoter of the *Mag* gene, an intronic enhancer of the *Aatk* gene or an upstream enhancer of the *Mbp* gene as examples of well-characterized regulatory regions with Sox10-dependent activity in differentiating oligodendrocytes (Figure 2B) (8,19). Whereas *Mag* and *Mbp* code for structural myelin proteins, *Aatk* is the host gene of *miR-338*, a differentiation promoting micro-RNA in oligodendrocytes (35).

Following transfection in N2a cells, all newly identified ECRs associated with OPC-expressed genes exhibited a robust Sox10-dependent activation in a range similar to the other regulatory regions (compare Figure 2C–E to F–J). No activation was observed when Sox10 was replaced by Sox6 as another OPC-expressed Sox protein (Figure 2C–J) (14). Regulatory regions from the *Mag, Aatk* and *Mbp* genes furthermore responded to the joint presence of Myrf and Sox10 in transfected cells with a substantially higher activity. For the *Mag* promoter, induction rates increased from 13 ± 3-fold in the presence of Sox10 to 39 ± 8-fold in the presence of Sox10 and Myrf (Figure 2H). For the *Aatk* regulatory region, induction rates increased from 34 ± 8 fold to 53 ± 4 fold and for the *Mbp* enhancer from 8 ± 1-fold to 14 ± 2-fold (Figure 2I, J). As previously reported, the presence of Myrf had little effect on its own (8), and Sox6 could not substitute for Sox10 (Figure 2H–J). This confirms the specific synergistic activation of CNS myelin genes by Sox10 and Myrf (8,9).

In contrast, regulatory regions from the newly identified OPC-specific Sox10 target genes exhibited lower activation rates in the presence of Sox10 and Myrf than in the presence of Sox10 alone with induction rates falling from 8 ± 2-fold to 2 ± 1-fold for *Tgfb2*, from 10 ± 2-fold to 3 ± 1-fold for *Id4* and from 19 ± 3-fold to 5 ± 1-fold for *Wnt7a* (Figure 2C–E). Intriguingly, a similar response towards Myrf was also observed for the intronic enhancer of the *Cspg4* gene and the *Pdgfra* promoter. In case of the *Cspg4* enhancer, Sox10-dependent activation rates fell from 37 ± 3-fold to 20 ± 3-fold, in case of the *Pdgfra* promoter from 4 ± 1-fold to 1-fold (Figure 2F, G). This suggests that Myrf may have a dual function in oligodendroglial cells. It synergistically supports Sox10 activity on its oligodendrocyte-specific target genes, but at the same time inhibits Sox10 function on its OPC-specific target genes.

### Myrf represses OPC-specific Sox10 target genes in primary oligodendrocytes and inhibits OPC characteristics

**Figure 3.**
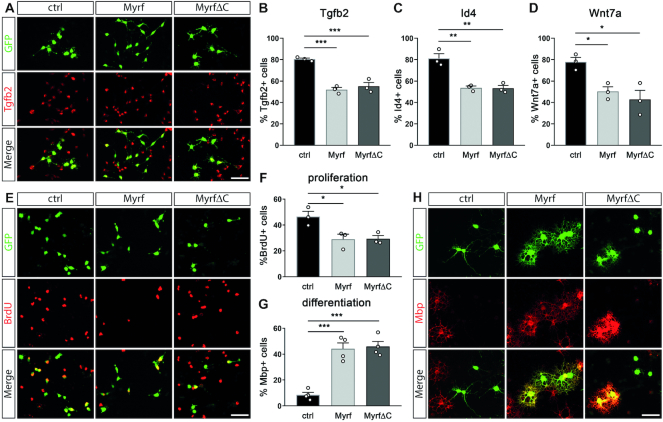
Effects of ectopic Myrf expression on transcription of target genes, proliferation and differentiation of OPCs. (**A**) Immunocytochemical detection of Tgfb2 (red) in primary rat oligodendroglial cells that were transduced with control (ctrl), Myrf or MyrfΔC expressing retrovirus. Transduced cells were visualized by virally encoded GFP (green). Tgfb2 and GFP are shown in separate channels and as merge. (**B–D**) Quantification of the fraction of transduced oligodendroglial cells that expressed Tgfb2 (B), Id4 (C) or Wnt7a (D). Transduction was with control, Myrf or MyrfΔC expressing retrovirus (*n* = 3). (**E**) BrdU incorporation (red) of primary oligodendroglial cells transduced with control, Myrf or MyrfΔC expressing retrovirus and kept under proliferative conditions. Transduced cells were visualized by virally encoded GFP (green). BrdU and GFP are shown in separate channels and as merge. (**F, G**) Quantification of the fraction of retrovirally transduced oligodendroglial cells that had incorporated BrdU (F, *n* = 3) under proliferative conditions or expressed Mbp under differentiating conditions (G, *n* = 4). (**H**) Immunocytochemical detection of Mbp (red) in primary oligodendroglial cells that were transduced with control, Myrf or MyrfΔC expressing retrovirus and kept for 3 days under differentiating conditions. Transduced cells were visualized by virally encoded GFP (green). Mbp and GFP are shown in separate channels and as merge. Scale bars: 50 μm. Differences to controls were statistically significant as determined by one way Anova with Bonferroni correction (*P ≤ 0.05; **P ≤ 0.01; ***P ≤ 0.001).

**Figure 4.**
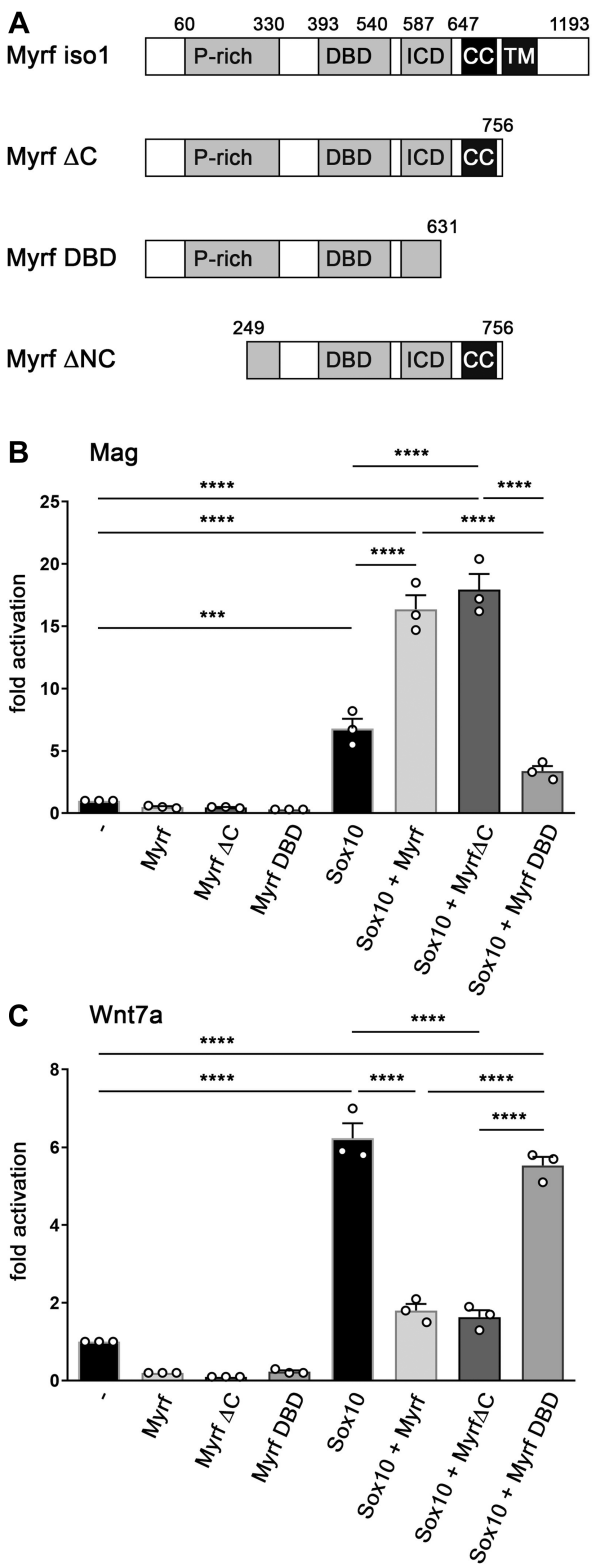
Structural requirements for Myrf function. (**A**) Schematic representation of the Myrf protein, its functional domains and the mutants used in the study. Numbers indicate positions of amino acids that define beginning or end of domains or constructs. P-rich, proline-rich domain; DBD, DNA binding domain; ICD, intracellular chaperone domain; CC, coiled coil domain; TM, transmembrane domain. (**B, C**) Luciferase assays in N2a cells transiently transfected with reporter genes under control of the *Mag* promoter (B) or *Wnt7a* regulatory region (C) in the absence (-) or presence of Myrf, various Myrf mutants, Sox10 and combinations thereof. Reporter gene expression was determined in extracts 48 h after transfection and effector-dependent activation rates are presented as fold inductions ± SEM with transfections in the absence of effectors arbitrarily set to 1 (*n* = 3). Differences were statistically significant as determined by one way Anova with Bonferroni correction (*P ≤ 0.05; **P ≤ 0.01; ***P ≤ 0.001).

To confirm the inhibitory effect of Myrf on the expression of OPC-specific Sox10 target genes, we transduced primary rat oligodendroglial cells in culture with a Myrf-expressing retrovirus. After another two days in culture, cells underwent fixation. Immunocytochemistry was employed for GFP to identify transduced cells and for Tgfb2, Id4 and Wnt7a to monitor their protein levels. Compared to mock-transduced control oligodendroglial cells, fewer Myrf-transduced cells were positive for Tgfb2, Id4 or Wnt7a (Figure 3A–D). A comparable reduction in Tgfb2, Id4 or Wnt7a expressing cells was also observed for cells transduced with a virus containing MyrfΔC (Figure 3A–D). This MyrfΔC protein lacks the carboxyterminal third (Figure 4A) that anchors newly synthesized Myrf in the ER membrane (9,10).

The Myrf-induced changes in expression of Tgfb2, Id4 or Wnt7a proteins went along with more general changes in oligodendroglial properties. When kept under proliferating conditions, Myrf-transduced oligodendroglial cells exhibited a lower proliferative capacity than their mock-transduced counterparts (Figure 3E, F). Under differentiating conditions, Myrf-transduced cells showed a higher propensity for differentiation. This was not only evident from an increased percentage of Mbp-expressing cells and higher Mbp levels per cell. The cells also covered a larger area and had more processes with a higher degree of ramification and greater portions of membrane sheets (Figure 3G, H). These morphological changes are characteristic of differentiating oligodendrocytes and suggest that Myrf-transduction indeed promotes oligodendrocyte differentiation rather than just premature Mbp expression. Again, transduced cells expressing MyrfΔC behaved similar to those expressing full length Myrf. These results confirm that Myrf has general effects on oligodendroglial properties by promoting differentiation and inhibiting proliferation. These effects furthermore appear largely mediated by the aminoterminal two thirds of the protein.

### The influence of Myrf on Sox10 activity depends on its ability to trimerize

To understand the mechanism that allows Myrf to support Sox10 on its oligodendrocyte-specific target genes and at the same time repress it on its OPC-specific target genes, we analyzed several Myrf mutants (Figure 4A) for their effects on Sox10-dependent activation of the regulatory regions from the *Mag* and *Wnt7a* genes as representative oligodendrocyte- and OPC-specific target genes. MyrfΔC was able to stimulate Sox10 activity on the *Mag* regulatory region and inhibit Sox10 activity on the *Wnt7a* regulatory region arguing that the aminoterminal two thirds of Myrf are sufficient for both activities (Figure 4B, C). In contrast, MyrfDBD, a Myrf mutant further shortened at its carboxyterminus than MyrfΔC (Figure 4A) was no longer capable of synergistically activating the *Mag* regulatory region (Figure 4B). MyrfDBD also failed to repress the Sox10-dependent activation of the *Wnt7a* ECR (Figure 4C). Similar to MyrfΔC, MyrfDBD contains the complete DNA-binding domain. However, it lacks a functional ICD that mediates trimerization as well as autoproteolysis of Myrf into an aminoterminal nuclear and a carboxyterminal ER-anchored part (9,10). Our results therefore point to the ICD and its trimerization function as essential for both the stimulating and repressing activities of Myrf.

### DNA-binding of Myrf requires two consensus motifs in a defined distance

From ChIP-Seq experiments, 5′-CTGGYAC-3′ had been identified as consensus motif for Myrf binding in the genome (9). To analyze whether DNA binding of Myrf is a prerequisite for its stimulatory and repressive activities, we searched for potential binding motifs in the *Tgfb2, Id4*, *Wnt7a*, *Aatk*, *Mag* and *Mbp* regulatory regions. By allowing up to one mismatch, we detected multiple such motifs in all regulatory regions. There was no obvious difference in localization of these motifs to each other or to potential Sox10 binding sites that would clearly distinguish regulatory regions with activity in OPCs from regulatory regions with activity in oligodendrocytes. Thus, bioinformatics argued for binding of Myrf to all regulatory regions, but failed to offer an explanation for the differential response towards Myrf.

So far, DNA binding characteristics of Myrf have not been studied biochemically. To better understand its mode of action, we initiated such an investigation by EMSA using conditions we recently established to visualize Myrf binding to DNA *in vitro* (25). To optimize our ability to detect Myrf, we used a MyrfΔNC variant that consists of DNA-binding domain, ICD and intervening sequences and thus contains all regions for proper carboxyterminal processing, trimerization and DNA-binding (Figure 4A).

**Figure 5.**
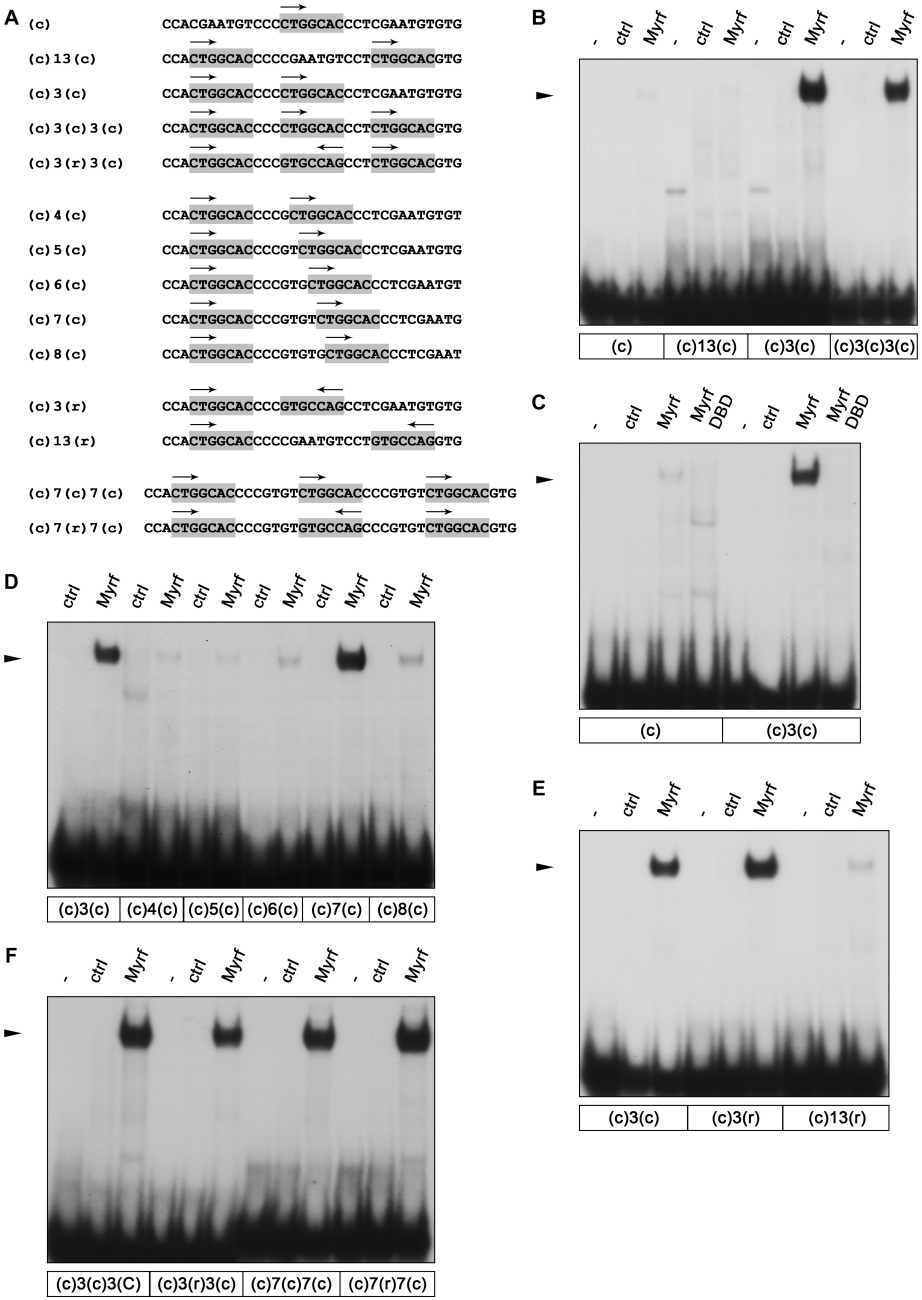
Effect of number, spacing and orientation of consensus motifs on the DNA binding ability of Myrf. (**A**) Sequence of oligonucleotides containing one or more consensus motifs for Myrf in different distances and orientations. Consensus motifs are highlighted by gray boxes; motif orientations are indicated by arrows. (**B–E**) EMSA with listed oligonucleotides as probes and extracts from HEK293 cells as protein source. HEK293 cells were transfected with empty (ctrl), MyrfΔNC (Myrf) or MyrfDBD (DBD) (see Figure 4A) expression plasmids. Analyzed was the influence of the number of consensus motifs (B), the requirement for Myrf trimerization (C), consensus motif spacing (D) and orientation (E) as well as a combination of number, spacing and orientation (F). –, no extract added. The position of the Myrf–DNA complex is marked by an arrowhead.

First, we analyzed whether Myrf would be able to bind to an oligonucleotide with a consensus 5′-CTGGCAC-3′ motif in the center (oligonucleotide (c) in Figure 5A). Contrary to our expectations from bioinformatic analysis, EMSA yielded only a very weak complex between Myrf and this oligonucleotide arguing against high-affinity binding (Figure 5B). Considering that Myrf forms trimers and that these trimers contain three DNA-binding domains, we reasoned that multiple consensus motifs may be necessary to permit high-affinity DNA-binding. Therefore, we designed oligonucleotides with two or three 5′-CTGGCAC-3′ motifs. The spacing was chosen such that the central nucleotides of each motif were 10 or 20 bp apart (Figure 5A). As a consequence, both motifs were separated by one or two helical turns and thus present on the same side of the DNA. The two motifs had identical head-to-tail orientation. An oligonucleotide with two 5′-CTGGCAC-3′ motifs spaced two helical turns apart again failed to bind substantial amounts of Myrf (oligonucleotide (c)13(c) in Figure 5B). In contrast, strong Myrf binding was observed to an oligonucleotide that contained the consensus motifs one helical turn apart (oligonucleotide (c)3(c) in Figure 5B). The presence of a third motif in head-to-tail orientation and one helical turn apart did not further increase Myrf binding (oligonucleotide (c)3(c)3(c) in Figure 5B). We conclude from these results, that at least two closely spaced 5′-CTGGCAC-3′ motifs are required to permit high-affinity binding and that the binding species is a Myrf trimer. The latter conclusion was further supported by EMSA with Myrf DBD. This monomeric Myrf variant failed to bind to DNA independent of whether oligonucleotides contained one or two consensus motifs in head-to-tail orientation and one helical turn apart (Figure 5C).

To further investigate the exact spacing requirements, we generated a set of oligonucleotides in which we changed the distance of the two consensus motifs by inserting up to five additional basepairs between them (Figure 5A). Already the addition of a single basepair dramatically reduced the ability of Myrf to bind (oligonucleotide (c)4(c) in Figure 5D). In fact, Myrf binding remained low on all oligonucleotides except the one with an insertion of four basepairs (oligonucleotide (c)7(c) in Figure 5D). This insertion changes the spacing of both motifs from one helical turn to one and a half turns. We therefore conclude that Myrf binding requires two consensus motifs that are either one helical turn apart and aligned on the same side of the helix, or one and a half turns apart on opposite sides.

To look into the effects of motif orientation, we also generated oligonucleotides with two consensus motifs spaced one or two helical turns apart and opposite (head-to-head) orientations (Figure 5A). In EMSA, these oligonucleotides behaved similar to the corresponding oligonucleotides that contained two consensus sites in identical orientations (Figure 5D). The one with a spacing of one helical turn between the two motifs was bound avidly (oligonucleotide (c)3(r) in Figure 5E), whereas the one with a spacing of two turns exhibited no substantial binding of Myrf (oligonucleotide (c)13(r) in Figure 5E). At first approximation, we conclude that the orientation of motifs relative to each other is of much less importance than their spacing.

With a better knowledge of the effects of motif orientation and spacing, we generated additional oligonucleotides with three consensus motifs in various orientations and spacings compatible with Myrf binding (Figure 5A) and compared Myrf binding to these oligonucleotides relative to the originally tested oligonucleotide where all three motifs had the same orientation and were spaced one helical turn apart. In our hands, Myrf bound all tested oligonucleotides equally well independent of orientation or spacing at one or 1.5 helical turns of the three consensus sites (Figure 5F). This reaffirms our previous conclusion that a third consensus motif has no further effect on high-affinity Myrf binding.

**Figure 6.**
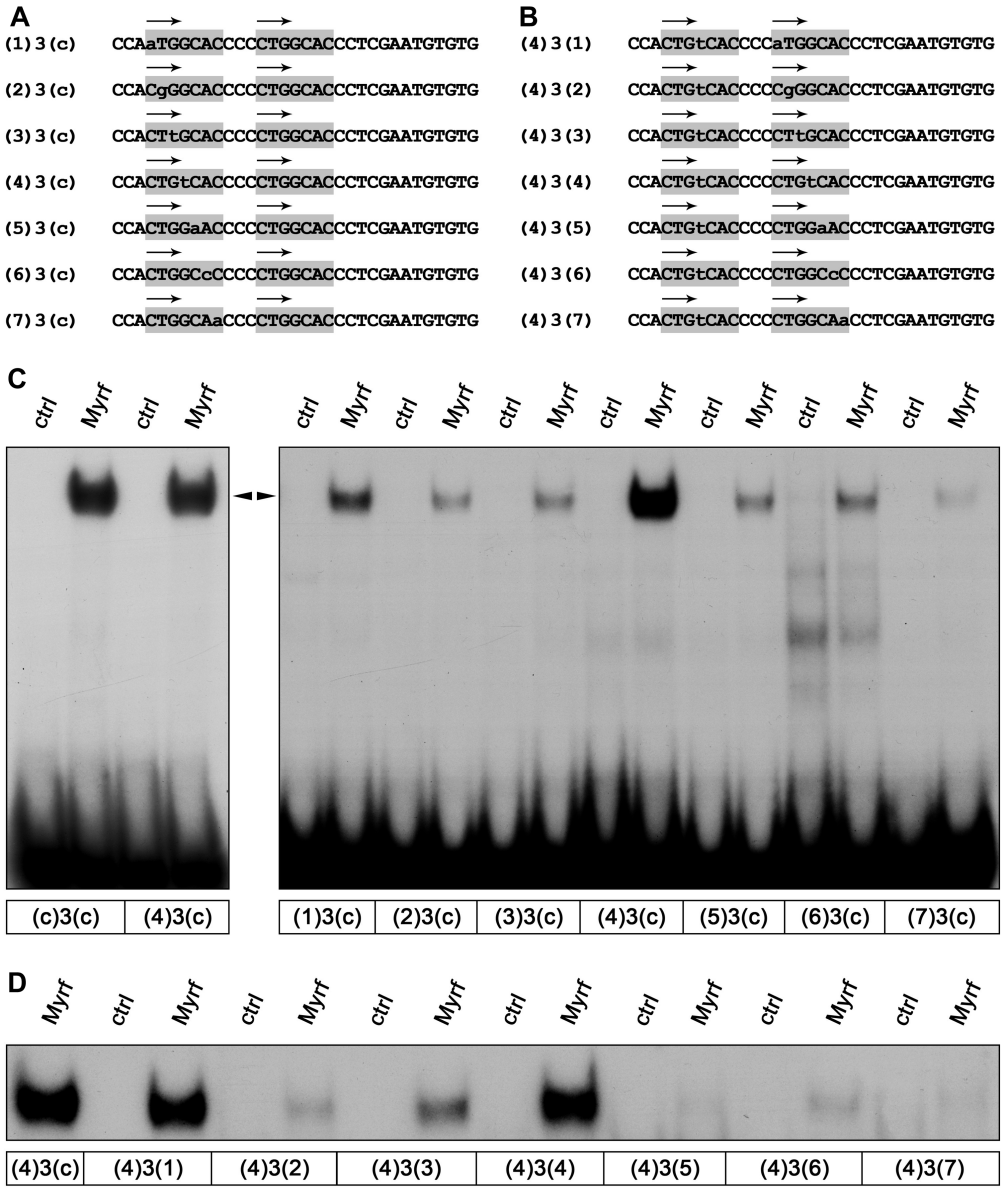
Effect of motif variations on DNA binding of Myrf. (**A, B**) Sequence of oligonucleotides containing two Myrf consensus motifs with single mismatches in the lateral motif (A) or with a mismatch at position 4 in the lateral motif and additional single mismatches in the central motif (B). (**C, D**) EMSA with oligonucleotides containing mismatches in one (C) or both (D) of the Myrf consensus motifs. Extracts from mock- (ctrl) or MyrfΔNC-transfected (Myrf) HEK293 cells served as protein source. The position of the Myrf–DNA complex is marked by an arrowhead.

To be able to predict genomic Myrf binding sites we next analyzed the impact of mismatches in one consensus motif on the ability of Myrf to bind. For that purpose, we used the oligonucleotide as template that contained two motifs in head-to-tail orientation spaced one helical turn apart, and generated a series in which single positions were exchanged in the lateral motif (Figure 6A). Changes were such that a purine was converted into the non-complementary pyrimidine and vice versa. In EMSA, most of the introduced mutations substantially reduced Myrf binding, but did not abolish it (Figure 6C). The impact on Myrf binding was particularly dramatic for the mutation introduced at position 7 (oligonucleotide (7)3(c) in Figure 6C) and less severe than most mutations for the change at position 1 (oligonucleotide (1)3(c) in Figure 6C). The mutation introduced at position 4 had no major effect on Myrf binding (oligonucleotide (4)3(c) in Figure 6C). We therefore conclude from these studies that a single mismatch in one of the two binding motifs may be compatible with Myrf binding depending on the exact nature of the altered basepair.

We next asked whether it would even be possible that both binding motifs contain a mismatch. For this set of experiments, we started out with the mutant oligonucleotide from the previous series that contained a mismatch at position 4 of the lateral motif, and now introduced additional mutations at single positions in the central motif employing the same principles as previously used in the study of the lateral motif (Figure 6B). As evident from EMSA, most introduced mutations severely affected Myrf binding (Figure 6D). However, some oligonucleotides retained a substantial amount of Myrf binding. Again changes at positions 1 and 4 appeared to be the least harmful (oligonucleotides (4)3(1) and (4)3(4) in Figure 6D). Our results therefore argue that Myrf may bind to two closely spaced motifs even if both do not fully conform to the consensus and have a mismatch. Our results also suggest that changes at positions 4 and 1 are least detrimental to Myrf binding, whereas changes at position 7 are most. However, these latter conclusions have to be taken with caution as we have introduced only one out of three possible changes at a particular position. Additionally, our binding motifs were embedded in a defined sequence environment. Both the exact nature of the introduced change and the surrounding sequences may have an impact on Myrf binding that we cannot predict from our studies.

### Transcriptional activation by Myrf requires two closely spaced consensus motifs

**Figure 7.**
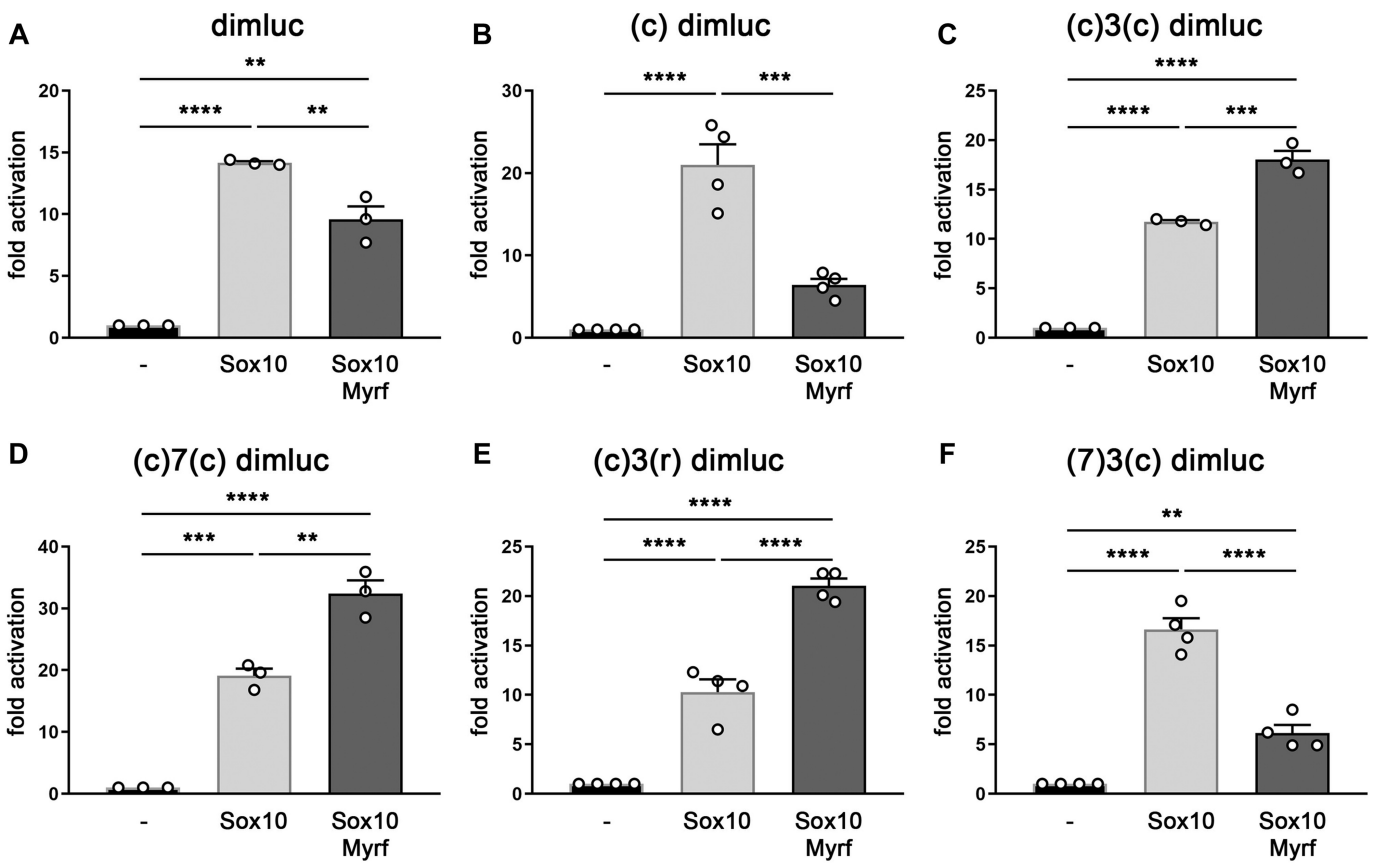
Effect of motif variations on transcriptional activity of Myrf. Reporter gene assays in transiently transfected N2a cells with dimluc-based reporters where luciferase is under control of a minimal promoter and Sox10 binding site C/C’. In addition to dimluc (**A**), several reporter variants were tested, in which the following Myrf binding sites were inserted: oligonucleotide (c) (**B**), oligonucleotide (c)3(c) (**C**), oligonucleotide (c)7(c) (**D**), oligonucleotide (c)3(r) (**E**) and oligonucleotide (7)3(c) (**F**). For sequences of oligonucleotides and Myrf binding abilities, see Figures 5 and 6. Transfections were carried out in the absence (-) or presence of Sox10 and Myrf as effectors. Reporter gene expression was determined in extracts 48 h after transfection and effector-dependent activation rates are presented as fold inductions ± SEM with transfections in the absence of effectors arbitrarily set to 1 (*n* = 3–4). Myrf did not change reporter gene expression substantially on its own (data not shown). Differences were statistically significant as determined by one way Anova with Bonferroni correction (*P ≤ 0.05; **P ≤ 0.01; ***P ≤ 0.001).

To analyze how the determined DNA-binding characteristics influence the transcriptional activity of Myrf, we generated a series of artificial reporter gene constructs. All of them contained a luciferase gene under control of a minimal promoter and a well-characterized dimeric Sox10 binding site (C/C’, see (23). As expected, this dimluc reporter construct was robustly activated by Sox10 in transiently transfected N2a cells (Figure 7A). When Myrf was additionally present in N2a cells, the Sox10-dependent activation decreased significantly. Into this reporter gene construct, we inserted several representative oligonucleotides from our binding studies. The integration of a single Myrf consensus motif in the luciferase reporter did not alter the behaviour of the construct. Again it was strongly activated in transiently transfected cells by Sox10 and much of the activity was lost when Myrf was additionally present (Figure 7B). However, when the fully functional Myrf binding site (c)3(c) was present that consisted of two consensus motifs at a distance of one helical turn, Sox10-dependent activation was increased in the presence of Myrf (Figure 7C). The same co-activation was observed when the consensus motifs were 1.5 helical turns apart, or in head-to-head instead of head-to-tail arrangement (Figure 7D, E). In contrast co-activation was lost when one of the two motifs contained a mismatch at position 7 (Figure 7F). Instead we observed a substantial reduction of the Sox10-dependent activation by Myrf with this reporter. These results argue that high-affinity binding of Myrf to a Sox10-responsive regulatory region likely correlates with co-activation.

### Myrf binds to oligodendrocyte-specific, but not to OPC-specific Sox10 target genes

**Figure 8.**
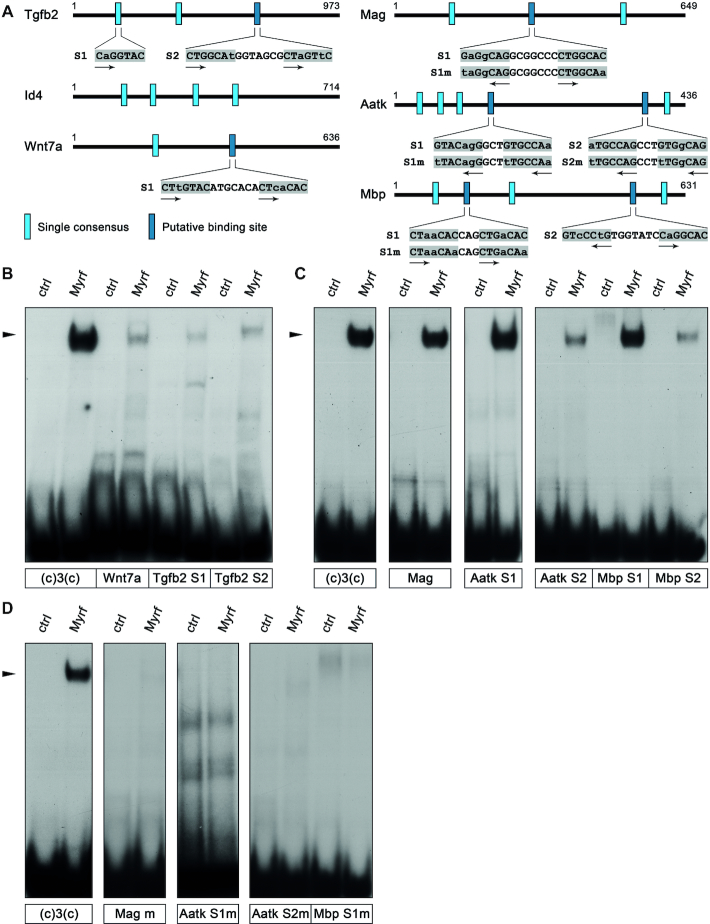
Identification of functional Myrf binding sites in the regulatory regions of Sox10 target genes. (**A**) Summary of potential Myrf binding sites with two or more consensus motifs in the regulatory regions of the *Tgfb2*, *Id4*, *Wnt7a*, *Mag*, *Aatk* and *Mbp* genes in naturally occurring (top) and mutated version (bottom). For the *Tgfb2* ECR, one of the many single consensus motifs is additionally shown. Consensus motifs are highlighted by gray boxes; motif orientations are indicated by arrows. (**B–D**) EMSA with oligonucleotides containing potential Myrf binding sites in OPC-specific (B) and oligodendrocyte-specific (C) Sox10 target genes or mutant versions thereof (D). Extracts from mock- (ctrl) or MyrfΔNC-transfected (Myrf) HEK293 cells served as protein source. The position of Myrf-DNA complexes is marked by an arrowhead.

After better understanding the DNA binding properties of Myrf, we revisited the regulatory regions of the *Tgfb2, Id4*, *Wnt7a*, *Mag, Aatk* and *Mbp* genes and searched for the presence of two potential binding motifs spaced 1–1.5 helical turns apart and with less than three mismatches to the consensus in total. This analysis revealed the presence of one potential Myrf binding site in the regulatory regions of the *Tgfb2*, *Wnt7a* and *Mag* genes and two potential ones in the regulatory regions of the *Aatk* and *Mbp* genes (Figure 8A).

Potential binding sites were analyzed as oligonucleotides in EMSA for their ability to bind Myrf. An oligonucleotide with a single consensus motif from the *Tgfb2* regulatory region was also included in the EMSA as a representative of the many single motifs in the various regulatory regions (Tgfb2 S1 in Figure 8A). However, no binding was observed to this single motif or any of the potential binding sites in the OPC-specific ECRs (Figure 8B). In contrast, strong binding of Myrf was observed to the Mag S1, the Aatk S1 and the Mbp S1 sites (Figure 8C). Weaker binding was detected to Aatk S2, followed by Mbp S2. Next, we introduced mutations into the Mag S1, Mbp S1, Aatk S1 and Aatk S2 sites (Figure 8A). These mutations abolished Myrf binding, thus confirming that binding sites were correctly determined (Figure 8D).

**Figure 9.**
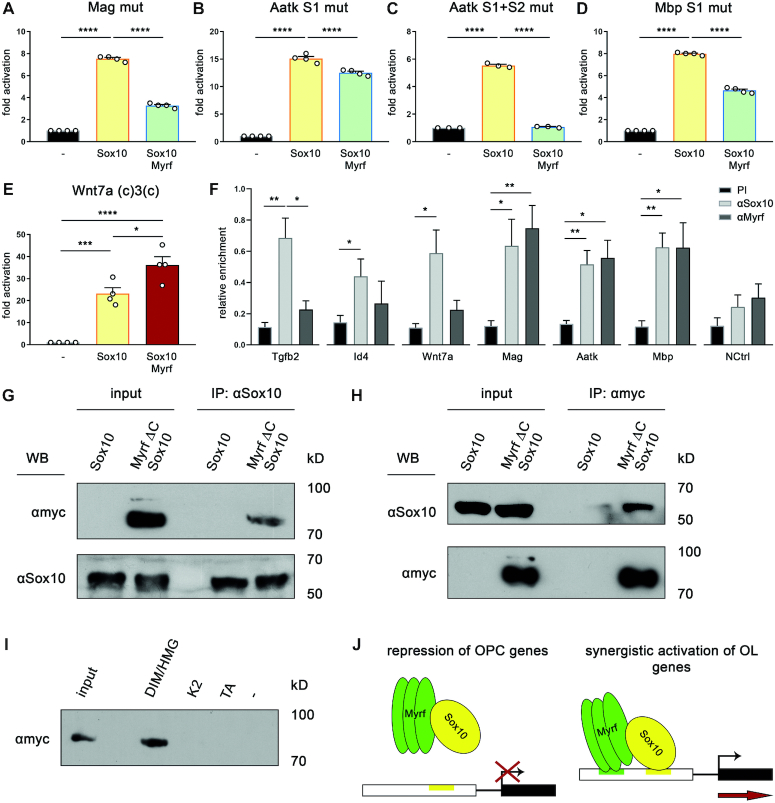
Functional and physical interaction between Myrf and Sox10. (**A–D**) Luciferase assays in N2a cells transiently transfected with reporter genes under control of regulatory regions from the *Mag* (A), *Aatk* (B, C) or *Mbp* (D) genes, in which Myrf binding sites were destroyed by mutation. (**E**) Luciferase assays in N2a cells transiently transfected with a reporter gene under control of the *Wnt7a* regulatory region in combination with Myrf binding site (c)3(c). Transfections were carried out in the absence (–) or presence of Sox10 and Myrf as effectors. Reporter gene expression was determined in extracts 48 h after transfection and effector-dependent activation rates are presented as fold inductions ± SEM with transfections in the absence of effectors arbitrarily set to 1 (*n* = 3–4). (**F**) ChIP on formaldehyde-crosslinked and sheared chromatin from primary rat oligodendroglial cells cultured for three days under differentiating conditions using rabbit pre-immune (PI, black bars), anti-Sox10 (αSox10, light gray bars), and anti-Myrf (αMyrf, dark gray bars) antisera. Amounts of immunoprecipitated chromatin were determined for the regulatory regions of the *Tgfb2*, *Id4*, *Wnt7a*, *Mag, Aatk* and *Mbp* genes and a negative control region (NCtrl) by quantitative PCR, and enrichments relative to input were calculated after normalization. Four independent immunoprecipitations were performed and the highest value of each experiment was set to 1. Presentation is as relative enrichment with mean values ± SEM. Differences were statistically significant as determined by two-tailed Student's t test (*, *P* ≤ 0.05; **, *P* ≤ 0.01; ***, *P* ≤ 0.001). (**G**) Co-immunoprecipitation of myc-tagged MyrfΔC with antiserum directed against Sox10 (αSox10) from HEK293 cell extracts that either contained Sox10 only or a combination of Sox10 and MyrfΔC. The upper Western blot was incubated with antibodies specific for the myc epitope, the lower with antibodies specific for Sox10. (**H**) Co-immunoprecipitation of Sox10 with antiserum directed against the myc epitope (αmyc) from HEK293 cell extracts that either contained Sox10 only or a combination of Sox10 and myc-tagged MyrfΔC. The upper Western blot was incubated with antibodies specific for Sox10 (αSox10), the lower with antibodies specific for the myc epitope. (**I**) GST-pulldown assays with myc-tagged MyrfΔC containing HEK293 extract (input) using bacterially expressed GST (-) or GST fused to specific domains of Sox10 (27), bound to glutathione sepharose beads as baits. The following Sox10 domains were used: DIM/HMG, dimerization and HMG-domain; K2, central protein-protein interaction domain; TA, transactivation domain. Bound MyrfΔC was visualized by Western blot using specific antibodies. Input corresponds to 1/20 of the amount of extract used in the assay. Numbers on the right side of western blots represent molecular weights of co-electrophoresed size markers. (**J**) Summary of proposed mode of Myrf action on OPC-specific and oligodendrocytes-specific target genes of Sox10.

When the same mutations were introduced into the context of the *Mag, Aatk* and *Mbp* regulatory regions, these regions retained their responsiveness to Sox10 when reporter gene assays were performed in transiently transfected N2a cells (Figure 9A–D). However, they were no longer synergistically activated by Myrf (compare Figure 9A–D to Figure 2H–J). Instead, Myrf now decreased the Sox10-dependent activation of the mutant *Mag, Aatk* and *Mbp* regulatory regions. This argues that synergistic activation of target genes by Sox10 and Myrf requires high-affinity binding of both proteins to the corresponding regulatory regions. In the absence of a binding site, Myrf impairs Sox10-dependent activation.

Myrf binding sites were not detected in the three analyzed regulatory regions from OPC-specific Sox10 target genes. When we artificially inserted such a binding site in immediate proximity to the *Wnt7a* regulatory region in a reporter plasmid, its behaviour changed dramatically (compare Figure 9E to Figure 2E). Myrf no longer repressed the Sox10-dependent activation of the *Wnt7a* ECR. Instead we obtained a synergistic activation by Sox10 and Myrf. We conclude that Myrf represses the activity of Sox10 on its OPC-specific target genes without binding to the corresponding regulatory regions.

To further confirm this conclusion, we analyzed in vivo binding of Sox10 and Myrf by performing ChIP on cultured oligodendroglial cells. After three days in differentiating conditions, cultures contain OPCs as well as oligodendrocytes. Using these cultures, *Tgfb2, Id4* and *Wnt7a* regulatory regions were enriched in chromatin precipitated with antibodies directed against Sox10 (Figure 9F). We assume that the precipitated chromatin stems from the OPCs present in the culture. No enrichment was obtained for any of the three regions after precipitation with antibodies directed against Myrf. ChIP therefore confirms that Myrf binding does not occur on the *Tgfb2, Id4* and *Wnt7a* regulatory regions in vivo. In contrast, *Mag*, *Aatk* and *Mbp* regulatory regions were equally enriched in chromatin precipitated with antibodies directed against Sox10 and antibodies directed against Myrf. (Figure 9F). Enriched chromatin likely stems from the fraction of differentiating oligodendrocytes in the culture. In line with our findings, overlapping peaks were also detected in published ChIP-Seq studies for Sox10 and Myrf in the *Aatk* and *Mbp* regulatory regions (9,20). For the *Mag* promoter only Myrf binding had been detected. Considering the Sox10 responsiveness of the *Mag* promoter and our ChIP results, we hypothesize that Sox10 binds as well and has been missed in the genome-wide study.

### Myrf inhibits Sox10 activity by sequestration

In the absence of binding sites for Myrf in Sox10-responsive regulatory regions, Myrf may physically interact with Sox10 and thereby prevent Sox10 from binding and activating these regions. To look into this possibility, we performed co-immunoprecipitation experiments. Previous co-immunoprecipitations had failed to detect an interaction between the aminoterminal part of Myrf and Sox10 (8). However, these experiments had used Myrf fragments that lacked the capability to trimerize.

By using HEK293 extracts that contained Sox10 and a myc-tagged version of the trimer-forming MyrfΔC protein (Figure 4A) we were able to precipitate MyrfΔC with Sox10 antibodies (Figure 9G) and Sox10 with myc-tag antibodies (Figure 9H). In GST pulldown experiments, MyrfΔC furthermore bound to a bacterially produced protein fragment spanning dimerization and DNA-binding domain of Sox10 (Figure 9I). No binding was detected to other known functional domains of Sox10 such as the centrally located protein-protein interaction domain K2 and the carboxyterminal transactivation domain. These results suggest that the aminoterminal two thirds of Myrf physically interact with Sox10 even in the absence of DNA-binding.

## DISCUSSION

Myrf is a central transcriptional regulator of the terminal differentiation process in oligodendrocytes and essentially required for CNS myelination (7). To fulfil its function, Myrf binds to regulatory regions of terminal differentiation and myelination genes and activates their expression (9). In this function, it cooperates with Sox10, another important transcription factor with multiple roles during several phases of oligodendroglial development (8,11,36).

Interestingly, Myrf induction precedes the induction of terminal differentiation, and reports of reduced survival of Myrf-deficient oligodendroglial cells during this short period suggest that Myrf is already required in oligodendroglial cells before the onset of terminal differentiation (7). Here, we present data that lead us to propose that during this period Myrf inhibits the activity of Sox10 on its target genes in OPCs and thereby helps to redirect Sox10 to a new set of target genes which it then synergistically activates with Myrf during terminal differentiation. We therefore suggest that Myrf acts as a modulatory switch for the activity of Sox10 (Figure 9J).

To unravel the underlying mechanism of this switch function, we first had to identify genes that are regulated by Sox10 in OPCs and the corresponding regulatory regions that mediate Sox10 activity. Additionally, we had to obtain a better understanding of the DNA-binding characteristics of Myrf. Bioinformatic analyses of ChIP-Seq data had determined that regions occupied by Myrf throughout the genome are enriched for 5′-CTGGYAC-3′ and had identified this heptameric consensus as binding motif for Myrf (9).

Our current studies, however, show that one consensus motif does not permit strong binding of Myrf. At least two motifs are required. This finding makes sense as Myrf is known to assemble into homotrimers in the ER-membrane before autoproteolysis and nuclear translocation of the aminoterminal part (9,10). Homotrimers of the aminoterminal part have been defined as the transcriptionally active species (25,37) and they possess three identical DNA-binding domains. We furthermore show in this study that the affinity of a single DNA-binding domain to consensus motifs is low. Therefore, our results indicate that two of the DNA-binding domains in the Myrf trimer have to contact consensus motifs to permit strong binding to DNA. Interestingly, we did not detect a further increase in binding strength when three consensus motifs were present. The reason for this is currently unknown. It may indicate that a DNA-binding domain in one of the Myrf subunits remains available for other functions, such as protein–protein interactions.

In accord with the well-defined, structurally tight arrangement of the DNA-binding domains in a trimer (38), binding motifs have to occur in a clearly defined distance to each other. We found that the two binding motifs had to be 1 or 1.5 helical turns apart. In contrast, binding site orientation was quite flexible with both head-to-tail and head-to-head arrangements equally functional. Similarly, we found that at least some mismatches in both binding motifs are compatible with continued high-affinity binding.

By applying this newly acquired knowledge to regulatory regions that are both responsive to Sox10 and Myrf, we were able to define two groups. One group of regulatory regions contained strong binding sites for Myrf and Sox10, mediated synergistic activation by both transcription factors and belonged to genes activated during terminal differentiation in oligodendrocytes. The other contained Sox10 binding sites, but lacked Myrf binding sites. These regulatory regions exhibited reduced Sox10-responsiveness in the presence of Myrf and segregated to genes that are activated by Sox10 in OPCs and downregulated upon terminal differentiation. This clearly argues that joint binding of Sox10 and Myrf is a prerequisite for synergistic activation. One possibility is that joint binding is cooperative and that cooperative binding triggers synergistic activation. Unfortunately, we cannot test this hypothesis for technical reasons. Myrf requires the use of polydIdC as unspecific competitor and is highly sensitive to polydGdC, whereas the opposite is true for Sox10. Therefore, we cannot perform EMSA with both proteins.

Myrf-dependent impairment of Sox10-responsiveness appears to follow a different mechanism. In this respect, it is important to note that the aminoterminal, homotrimerizing two thirds of Myrf physically interact with Sox10 even in the absence of DNA. Therefore it seems plausible that Myrf exerts its inhibitory role on the Sox10-dependent activation of regulatory regions from OPC genes by sequestering Sox10 away or redirecting it to regulatory regions that contain Sox10 and Myrf sites. This appears physiologically meaningful as it allows Sox10 to switch in a temporally controlled manner from one set of target genes to a different one as a prerequisite for proper lineage progression and successful terminal differentiation.

With this study, the regulatory relationship between Sox10 and Myrf has gained additional complexity. First, Sox10 induces Myrf which redirects Sox10 activity away from its OPC-specific target genes and then cooperates with Sox10 to activate a new set of genes required during terminal differentiation and myelination.

## DATA AVAILABILITY

All data generated or analyzed during this study are included in this published article or have been deposited in the GEO database (accession number GSE136659).

## Notes

Present address: Matthias Weider, Zahnklinik 3 – Kieferorthopädie, Universitätsklinikum Erlangen, Friedrich-Alexander-Universität Erlangen-Nürnberg, Erlangen, Germany.
